# Efficacy of the nano curcumin supplementation on fibrosis, steatosis, inflammatory and metabolic status of liver fibrosis patients with non-alcoholic fatty liver origin: a study protocol for a double-blind randomized controlled trial

**DOI:** 10.1186/s40795-026-01252-0

**Published:** 2026-02-02

**Authors:** Hadis Gerami, Hassan Mozaffari-Khosravi, Asieh Mansour, Amir Ali Sohrabpour, Hossein Poustchi, Amir Pejman Hashemi Taheri, Mahmoud Reza Jaafari, Sara Jambarsang, Sayyed Saeid Khayyatzadeh

**Affiliations:** 1 Department of Nutrition, School of Public Health, Shahid Sadoughi University of Medical Sciences, Yazd, 8914715645 Iran; 2 Center for Food Hygiene and Safety, School of Public Health, Shahid Sadoughi University of Medical Sciences, Yazd, Iran; 3 Endocrinology and Metabolism Research Center, Endocrinology and Metabolism Clinical Sciences Institute, Tehran University of Medical Sciences, Tehran, Iran; 4 Tehran Gastroenterology and Hepatology Centre (Masoud Clinic), Tehran, Iran; 5 Liver and Pancreatobiliary Diseases Research Center, Digestive Diseases Research Institute, Tehran University of Medical Sciences, Tehran, Iran; 6 Department of Radiology, School of Medicine, Shariati Hospital, Tehran University of Medical Sciences, Tehran, Iran; 7 Nanotechnology Research Center, Pharmaceutical Technology Institute, Mashhad University of Medical Sciences, Mashhad, Iran; 8 Department of Pharmaceutical Nanotechnology, School of Pharmacy, Mashhad University of Medical Sciences, Mashhad, Iran; 9 Center for Healthcare Data Modeling, Department of Biostatistics and Epidemiology, School of Public Health, Shahid Sadoughi University of Medical Sciences, Yazd, Iran

**Keywords:** Dietary supplements, Fibrosis, Random allocation, Clinical trial, Iran

## Abstract

**Background:**

Liver disease related to alcohol consumption and non-alcoholic fatty liver disease (NAFLD) are among the leading causes of liver fibrosis. Curcumin, due to its anti-fibrotic and anti-inflammatory effects, has been proposed as a potential therapeutic agent. However, its clinical application is limited by poor stability, solubility, and bioavailability. Nanocurcumin, a nano-formulated version of curcumin, overcomes these limitations. This study aims to evaluate the efficacy of Nanocurcumin supplementation in improving fibrosis, steatosis, inflammation, and metabolic status in patients with liver fibrosis of NAFLD origin.

**Methods:**

This double-blind, randomized, placebo-controlled trial is being conducted at the Gastroenterology Clinic of Shariati Hospital (Tehran, Iran). Fifty patients aged 30 to 70 years are enrolled and randomly assigned to the intervention or placebo group using a block balanced randomization method. The intervention group receives one capsule of Nanocurcumin (40 mg, twice daily), while the placebo group receives an identical placebo capsule. The primary outcomes include liver fibrosis and steatosis, assessed via FibroScan at baseline and at the end of the study. Secondary outcomes include changes in inflammatory markers, metabolic parameters, liver function tests, anthropometric measures, and blood pressure over the 16-week intervention period.

**Discussion:**

This trial is currently ongoing. Recruitment is expected to be completed by the end of 2024, and the trial is anticipated to be fully completed by mid-2025. If Nanocurcumin supplementation demonstrates positive efficacy, it may serve as a potential therapeutic approach for patients with liver fibrosis.

**Trial registration:**

Iranian Registry of Clinical Trials IRCT20210427051098N2 (Available from: https://irct.behdasht.gov.ir).

Date and version identifier: April 20, 2023; approved version by the IRCT.

**Supplementary Information:**

The online version contains supplementary material available at 10.1186/s40795-026-01252-0.

## Background

Non-alcoholic fatty liver disease (NAFLD) is an increasingly prevalent condition, closely associated with modern lifestyle factors such as poor diet, obesity, and sedentary behavior [1]. It encompasses a spectrum of liver conditions, ranging from simple steatosis (fatty liver) to non-alcoholic steatohepatitis (NASH), which can progress to liver fibrosis, cirrhosis, and ultimately liver failure or hepatocellular carcinoma [2]. The liver’s crucial role in metabolism, detoxification, and immune modulation makes it particularly vulnerable to metabolic imbalances and inflammatory processes inherent in NAFLD [3].

Liver fibrosis is a key factor in the progression of NAFLD to more severe forms. It is characterized by excessive accumulation of extracellular matrix proteins, particularly collagen, leading to scarring and impaired liver function [4]. This process is driven by chronic liver injury and inflammation, which activate hepatic stellate cells (HSCs) to transform into fibrogenic myofibroblasts [5, 6]. Despite the significant global health burden posed by liver fibrosis, current therapeutic options are limited, and liver biopsy remains the gold standard for diagnosis despite its invasive nature and associated risks [7–9].

Current treatment strategies for liver fibrosis primarily focus on lifestyle modifications, such as weight loss and dietary changes, which can effectively halt or reverse fibrosis in some patients [10, 11]. However, adherence to these lifestyle interventions is often poor, necessitating the development of pharmacological approaches. Although anti-fibrotic drugs have shown potential in preclinical studies, there is still a need for effective and safe therapies that can be translated into clinical practice [12–14].

Curcumin’s multi-faceted therapeutic properties make it a promising candidate for liver fibrosis treatment [15]. Studies have demonstrated its ability to modulate various molecular pathways involved in inflammation, oxidative stress, and fibrogenesis [16–18]. However, the clinical translation of curcumin has been limited by its pharmacokinetic challenges. Nanotechnology-based approaches offer a potential solution by enhancing curcumin’s bioavailability and improving its targeted delivery to the liver [19, 20].

Emerging research highlights the potential of curcumin, a polyphenolic compound derived from turmeric, in managing various health conditions. Its therapeutic effects are primarily attributed to its anti-inflammatory, antioxidant, and anti-fibrotic properties. However, curcumin’s clinical application is hindered by its poor bioavailability, rapid metabolism, and low solubility in bodily fluids [21–23]. To overcome these limitations, nanotechnology has been employed to develop Nanocurcumin formulations, which enhance curcumin’s stability, solubility, and bioavailability. Nano carriers such as liposomes, polymer micelles, and solid lipid nanoparticles have shown promise in improving the delivery and efficacy of curcumin in various therapeutic contexts [19, 20].

This study aims to bridge the gap in clinical evidence regarding the efficacy of Nano curcumin in treating liver fibrosis of NAFLD origin. By conducting a randomized controlled trial, we aim to generate data on the therapeutic potential of Nanocurcumin. This evidence will help inform clinical practice and guide the development of effective treatment strategies for liver fibrosis. The primary objective of this study is to evaluate the efficacy of Nanocurcumin supplementation in the treatment and slowing of liver fibrosis and steatosis progression in patients with NAFLD-related fibrosis. Secondary objectives include assessing changes in inflammatory markers, metabolic parameters, liver function tests, anthropometric measures, and blood pressure. The findings from this study will contribute to a better understanding of Nanocurcumin’s role in managing liver fibrosis and may support its potential as a therapeutic option for patients with NAFLD-related fibrosis.

## Methods

### Trial design

This study is a parallel, randomized, double-blind, placebo-controlled trial (IRCT20210427051098N2), which was approved by the institutional review board of the Shahid Sadoughi University of Medical Sciences (SSUMS, Yazd, Iran) and research ethics committee (approval ID: IR. SSU. SPH.REC. 1401. 157) on January 29, 2023. This protocol has been written according to the SPIRIT2013 (Standard Protocol Items: Recommendations for Interventional Trials) checklist (Supplementary) [24].

### Setting

This study is conducted at the Gastroenterology and Liver Clinic of Shariati Hospital affiliated with Tehran University of Medical Sciences (Tehran, Iran). It should be noted that this clinic serves as a major referral center for liver diseases within the region.

### Eligibility criteria

Participants are adults aged 30 to 70 years are included in the study according to the following criteria (Table 1). Participants will be studied after obtaining the written informed consent in Farsi language (Supplementary) by the principle investigator of the study.

**Table 1:** Participants eligibility criteria

**Inclusion criteria**: • Stage of Liver Fibrosis: Individuals with liver fibrosis stage ≥ F2. • Diagnosis: Liver fibrosis patients with the origin of non-alcoholic fatty liver disease (NAFLD) and referred to the Gastroenterology Clinic of the Shariati Hospital affiliated with TUMS (Tehran, Iran). **Exclusion Criteria**: • Substance use: Individuals with a history of alcohol addiction, current use of alcohol, or substance abuse problems. • Other liver diseases: Patients with other chronic or acute liver conditions, such as hepatitis B, hepatitis C, biliary diseases, autoimmune liver diseases, or genetic disorders affecting liver metabolism (e.g., hemochromatosis or Wilson’s disease). • Pregnancy and lactation: Women who are pregnant or breastfeeding. • Medication use: Individuals taking hepatotoxic drugs or undergoing treatment with anti-inflammatory drugs, corticosteroids, or hormones. • Serious comorbid conditions: Individuals with severe lung or kidney disease, or active cancer. • Dietary and medication changes: Patients who are on weight loss diets, have recently changed their medication regimen, or are using supplements that could interfere with the study. • Warfarin therapy: Individuals undergoing warfarin therapy due to the increased risk of bleeding and the potential for interaction with Nanocurcumin. • Participation willingness: Those unwilling to participate or adhere to the study protocol. • Side effects of Nanocurcumin supplement: Patients who experience gastrointestinal complications after taking nanocurcumin.	

### Intervention and control group

In this study, patients in the intervention group receive one capsule of Nanocurcumin supplement (40 mg, twice a day), while patients in the placebo group receive one placebo capsules twice a day. These placebo capsules are designed to be similar to the Nanocurcumin capsules in terms of appearance, color, and smell. Both the Nanocurcumin and placebo capsules are prepared by Exir Nano Sina (ENS) Company in Iran, using Maltodextrin in the placebo capsules. Sufficient quantities of capsule-containing bottles are delivered to patients at the beginning of the study and at the end of weeks 4, 8, 12, and 16. It is recommended that patients consume the capsules with a main meal and cold water. Nausea, Vomiting and Diarrhea are the common side effects of Curcumin when used as powder. However, incidence of these conditions using nanomicellar Curcumin is significantly decreased. Additionally, dietary and physical activity recommendations are provided to the patients in both groups.

### Outcomes and measurements

#### Primary outcomes

##### Assessment of liver fibrosis and steatosis

It is measured using transient elastography by fibroscan (Echosens, Paris, France) [25, 26] at baseline and at the end of the study (16 months). Evaluation of liver fat accumulation and scarring The samegastroenterologist will use transient elastography with fibroscan to investigate hepatic fibrosis and steatosis in all patients at the beginning and end of the trial. Patients will be instructed to assume a supine position and position their right hand above their head. Next, the probe will be positioned on the right side of the liver in the intercostal area of the patients. The measurement of liver fibrosis will be reported in Kilopascal (Kpa). The fibrosis assessment results will be interpreted based on the METAVIR Score.

The stages are as follows:

- F0: no fibrosis
- F1: mild fibrosis, but liver structure and function are still normal
- F2: portal fibrosis with few septa
- F3: numerous septa without cirrhosis
- F4: cirrhosis, which is permanent scarring and liver disease has progressed to the final stage [27].

The controlled attenuation parameter (CAP) test will be utilized for evaluating steatosis, with the outcomes being expressed in decibels per meter (dB/m). The CAP findings will range from 100 to 400 dB/m, FibroScan is a non-invasive technique that utilizes ultrasound technology to assess liver stiffness and fat buildup. It offers dependable information on fibrosis and steatosis, eliminating the necessity for a liver biopsy [28].

#### Secondary outcomes

##### Inflammatory markers

Levels of key inflammatory cytokine including high sensitivity c reactive protein (hs-CRP) and erythrocyte sedimentation rate (ESR) will be quantified using Enzyme-linked immunosorbent assay (ELISA) assays from blood samples collected at baseline and at the end of the study.

##### Metabolic status

Changes in metabolic parameters, including fasting plasma glucose (FPG), Insulin, lipid profile including, total cholesterol (TC), low-density lipoprotein cholesterol (LDL-C), high-density lipoprotein cholesterol (HDL-C) and triglycerides (TG), will be measured using an ELISA kit at baseline and at the end of the 16-month period.

##### Liver factors and other biochemical assessment

These include alanine Aminotransferase (ALT), aspartate aminotransferase (AST), alkaline phosphatase (ALP), lactate dehydrogenase (LDH), total bilirubin (T-Bil), direct bilirubin (D-Bil) and gamma-glutamyl transferase (GGT) levels, Albumin (ALB) and Platelets (PLT) which will be measured using standard biochemical methods at baseline and at the end of the study to assess liver function.

##### Anthropometric measures

In order to measure height accurately, the individual should be in an upright position and without any footwear, with a precision of 0.5 cm. Weight will be assessed using a digital scale while wearing minimal clothing and being barefoot, with a precision of 100 g. The waist circumference (WC) will be assessed by employing a tape measure positioned precisely between the supra iliac bone and the final rib, with a precision of 0.5 cm. body mass index (BMI) can be calculated by dividing the weight in kilograms by the square of the height in meters.

##### Blood pressure

The patient’s systolic blood pressure (SBP) and diastolic blood pressure (DBP) will be assessed using a mercury sphygmomanometer. This will be done after the patient has rested for 5 min while sitting, and the measurements will be taken from their right hand. Prior to blood pressure examination, patients should abstain from consuming alcohol, engaging in physical activity, or smoking for a duration of one hour. Prior to testing their blood pressure, it is necessary for individuals to ensure that their bladder is empty. The patient’s blood pressure will be measured twice, with a minimum time interval, while they are in a resting state. Systolic blood pressure corresponds to the pressure recorded immediately after the detection of the initial korotkoff sound, whereas diastolic blood pressure corresponds to the pressure recorded when the korotkoff sound ceases. The average of the two values will be regarded as the systolic and diastolic blood pressure.

##### Dietary recall and physical activity

To maintain consistency in the participants’ food intakes throughout the trial, we will gather information on their nutritional intake by conducting a 24-hour dietary recall, 3 days at the beginning of the study and 3 days at the end of the study. The distribution of these dietary recalls will occur on weekdays to encompass four for workdays and two weekends. The reported foods in these recalls will be converted to grams using the available booklets. The analysis will assess the average of intake based on six 24-hour dietary recalls for each participant. Participants will be instructed to maintain their current level of physical activity during the study. In order to ensure accuracy, we will complete Physical activity questionnaire at the beginning and The end of the study for each participant based on the International Scientific Activities Questionnaire (IPAQ) [29]. To analyze the physical activity data, we will utilize previously established criteria that provide the Metabolic equivalent of task- hour/day (MET-h/day) values for different types of physical activities. These guidelines take into account the duration of each participant’s engagement in a specific physical activity.

Both groups will receive general dietary and physical activity recommendations to ensure consistency and minimize confounding factors. However, no specific dietary modifications will be imposed that could independently influence fibrosis, steatosis, inflammation, or metabolic status.

#### Additional outcomes

##### Adherence and tolerability

These are monitored through patient diaries where capsule intake and any adverse effects are recorded daily. If any participant experiences symptoms or other intolerances during the study, they will have the option to withdraw from the intervention. Compliance will be further verified during biweekly telephone check-ins and by counting returned capsules at monthly visits.

### Participant timeline

Time schedule of enrolment, intervention, and outcomes assessments is shown in Table 2.

**Table 2:** The schedule ofenrolment, interventions, and assessments*

	Study Period			
	Enrolment	Allocation	Post-allocation (16-weeks)	Close-out(at the end of 16 weeks)
Timepoint**	***-t***_***1***_	**0**	***T1-T16***	
Enrolment:				
Eligibility criteria screening	X			
Informed consent obtaining	X			
Allocation		X		
**Intervention**			X	
Nano curcumin supplementation			X	
Placebo			X	
**Assessments**				
Baseline measurements	X			X
Primary outcome	X			X
Secondary outcomes	X			X
Additional outcomes	X			X

### Sample size

The sample size is determined based on the study primary outcome (i.e., liver fibrosis). It was calculated using G*Power version 3.1.9.2 using the values from the previous study [30]. Therefore, by considering the effect size of 0.9, type I error (α) of 5% and statistical power (β) of 80%, and an attrition rate of 10%, the total sample size was calculated to be 50 patients, with 25 patients allocated to each of group.

### Randomization and blinding

Patients are randomly assigned to two groups using a block balance randomization. Both the intervention and control groups receive supplements, which are manufactured by Exir Nano Sina (ENS) Company in Iran, and designed to be indistinguishable in shape, color, size, and packaging to maintain blinding. A third party, not involved in the research, labels the containers with either “A” or “B,” which keeps the researchers blind to the group assignment.

### Data management

Baseline and end-of-study assessments encompass a comprehensive range of tests. A total of 14 mL of blood will be collected at baseline and at the end of the study. The blood will be divided into separate aliquots to ensure accurate biochemical analyses of inflammatory markers, metabolic parameters, and liver function tests. Anthropometric measurements including weight, height, waist circumference, and blood pressure, are recorded by a research nutritionist. Liver fibrosis and steatosis levels are evaluated using FibroScan at the start and end of the study.

### Data collection

Data will be collected at specified times throughout the study, namely at baseline and at the end of the study. The research staff will be receiving training on correct data collection, entry, and management techniques to ensure data accuracy and reliability.

### Data recording

Data will be recorded on paper. Each participant will be assigned a unique identification to anonymizes their personal information and maintain confidentiality. The paper documents will be stored in a secure area with limited access.

### Data entry and verification

Data entry will be conducted by qualified individuals using a database specifically created for this project. To minimize errors, essential variables such as liver stiffness measures and biochemical indicators will be entered twice. Any inconsistency between the entries will be addressed by cross-referencing with the original paper records.

### Data storage

All paper forms will be securely stored at Shariati Hospital. Access to the data will be limited to authorized study personnel only, and all access will be logged to ensure traceability.

### Quality assurance

Regular audits of the data will be conducted to ensure ongoing accuracy and completeness. These audits will be performed by independent members of the research team who are not directly involved in data collection. Any issues identified during audits will be addressed promptly to maintain the integrity of the data.

### Data confidentiality

To protect participant confidentiality, all personal identifiers will be removed from the study data and replaced with unique identification numbers. Only the principal investigator and designated members of the research team will have access to the key that linking these numbers to participant identities.

### Data sharing and long-term storage

Data will be securely archived for at least 10 years after the study is completed, in accordance with institutional and ethical norms. Summary data can be disseminated to the broader scientific community through publications and presentations at scientific events. Requests for access to de-identified participant data for valid scientific research will be reviewed by the study’s steering committee in accordance with ethical and regulatory guidelines.

### Statistical analysis

In this study, data is statistically analyzed using SPSS version 24 software. The Chi-Square test is utilized to compare categorical variables between two groups. Quantitative data will be assessed for normality, and the data with normal distribution will be compared using an independent sample t-test. Alternatively, the Mann-Whitney U test will be employed for non-normally distributed data. For outcome analysis, the ANOVA/ANCOVA will be used. A statistical significance level of *P* < 0.05 will be considered.

## Results

This trial is currently ongoing. Recruitment is expected to be completed by the end of 2024, and the trial is anticipated to be fully completed by mid-2025.

## Discussion

In the present study, we evaluated the efficacy of nano curcumin supplementation on liver fibrosis patients with non-alcoholic fatty liver origin. Liver fibrosis, a major public health concern, significantly impacts how liver disease progresses and raises the risk of hepatocellular carcinoma (HCC). Studies show that if the cause of liver fibrosis is addressed, the scarring can be reversed. This highlights the urgent need for new medications (anti-fibrotic therapies) to prevent liver fibrosis from worsening and to stop HCC development. However, despite promising results in animal studies, many potential anti-fibrotic drugs have been absent or limited in human trials [31]. Nano curcumin, with its enhanced bioavailability and anti-inflammatory and antioxidant properties, presents a promising therapeutic option for liver fibrosis [2].

Curcumin’s poor absorption has historically limited its therapeutic potential in treating liver fibrosis. The development of nanocurcumin formulations aims to overcome these limitations and enhance its therapeutic efficacy [32]. Previous studies have demonstrated curcumin’s ability to reduce liver enzymes and improve histological markers of liver health, which are promising for treating liver fibrosis and NAFLD [33, 34]. This study builds on these findings by using a nano-formulated version of curcumin to explore its potential for providing meaningful clinical benefits.

However, it is important to note that while extensive research has been conducted on the anti-fibrotic effects of curcumin in molecular and laboratory phases, there is limited work in the human phase. Specifically, as far as we know, no studies have focused on the Nano curcumin format in patients with liver fibrosis. Some research involving curcumin or Nano curcumin has been conducted on fatty liver patients, some of whom had fibrosis. Our study uniquely targets patients with liver fibrosis exclusively, thereby providing focused insights into this subgroup.

Among the many challenges in running and overseeing a clinical trial involving dietary supplements such as Nano curcumin are making sure participants follow the supplementation regimen and the strict follow-up timetable required to evaluate long-term effects on liver fibrosis and steatosis. Assessing the actual efficacy of Nano curcumin requires the placebo-controlled, double-blind methodology, but maintaining blinding and addressing possible biases requires careful implementation.

Although our study is the first to evaluate the effectiveness of nanocurcumin supplementation in liver fibrosis over a relatively appropriate timeframe, it has certain limitations. First, this is small-scale trial, which may restrict the generalizability of the findings. Second, the study population is limited to patients with liver fibrosis of NAFLD origin, potentially excluding other subsets of liver fibrosis, such as those caused by alcoholic liver disease or other etiologies. Third, many patients with NAFLD often present with comorbidities such as obesity, diabetes, and metabolic syndrome, which could confound the treatment outcomes and limit the ability to isolate the effects of the intervention.

The findings of this study will contribute to the growing body of evidence supporting Nanocurcumin as a potential therapeutic option for liver fibrosis. However, further large-scale studies with longer follow-up time are needed to explore long-term benefits, optimal dosing strategies, and potential interactions with other treatments of Nanocurcumin.

If the trial demonstrates positive effects on fibrosis and steatosis, it would provide a basis for further investigation in larger, multicenter studies. In conclusion, Nanocurcumin supplementation is being evaluated as a potential intervention for liver fibrosis; however, additional research is required before it can be considered for clinical application.

## Supplementary Information

Supplementary Material 1.

Supplementary Material 2.

## Data Availability

The datasets will be available from the corresponding author on reasonable request after finishing the study.

## Abbreviations

NAFLD: Non-alcoholic fatty liver disease
NASH: Non-alcoholic steatohepatitis
HSCs: Hepatic stellate cells
ENS: Exir Nano Sina
CAP: Controlled attenuation parameter
hs-CRP: High sensitivity c reactive protein
ESR: Erythrocyte sedimentation rate
ELISA: Enzyme-linked immunosorbent assay
FPG: Fasting plasma glucose
TC: Total cholesterol
LDL-C: Low-density lipoprotein cholesterol
HDL-C: High-density lipoprotein cholesterol
TG: Triglyceride
ALT: Alanine Aminotransferase
AST: Aspartate aminotransferase
ALP: Alkaline phosphatase
LDH: Lactate dehydrogenase
T-Bil: Total bilirubin
D-Bil: Direct bilirubin
GGT: Gamma-glutamyl transferase
ALB: Albumin
PLT: Platelets
WC: Waist circumference
BMI: Body mass index
SBP: Systolic blood pressure
DBP: Diastolic blood pressure
IPAQ: International Scientific Activities Questionnaire
MET-h/day: Metabolic equivalent of task- hour/day
HCC: Hepatocellular carcinoma

## References

[1] Chen Z, Liu J, Zhou F, Li H, Zhang X-J, She Z-G, et al. Nonalcoholic fatty liver disease: an emerging driver of cardiac arrhythmia. Circul Res. 2021;128(11):1747–65. 10.1161/CIRCRESAHA.121.319059 34043417

[2] Mansour A, Mohajeri-Tehrani MR, Samadi M, Gerami H, Qorbani M, Bellissimo N, et al. Risk factors for non-alcoholic fatty liver disease-associated hepatic fibrosis in type 2 diabetes patients. Acta Diabetol. 2019;56:1199–207. 31197470 10.1007/s00592-019-01374-x

[3] Elpek GÖ. Cellular and molecular mechanisms in the pathogenesis of liver fibrosis: an update. World J Gastroenterology: WJG. 2014;20(23):7260. 10.3748/wjg.v20.i23.7260 PMC4064072 24966597

[4] Tacke F, Weiskirchen R. Non-alcoholic fatty liver disease (NAFLD)/non-alcoholic steatohepatitis (NASH)-related liver fibrosis: mechanisms, treatment and prevention. Annals Translational Med. 2021;9(8):729. 10.21037/atm-20-4354 PMC8106094 33987427

[5] Jiang JX, Török NJ. Liver injury and the activation of the hepatic myofibroblasts. Curr Pathobiology Rep. 2013;1:215–23. 10.1007/s40139-013-0019-6 PMC3748972 23977452

[6] Dewidar B, Meyer C, Dooley S, Meindl-Beinker N. TGF-β in hepatic stellate cell activation and liver fibrogenesis—updated 2019. Cells. 2019;8(11):1419. 31718044 10.3390/cells8111419 PMC6912224

[7] Heyens LJ, Busschots D, Koek GH, Robaeys G, Francque S. Liver fibrosis in non-alcoholic fatty liver disease: from liver biopsy to non-invasive biomarkers in diagnosis and treatment. Front Med. 2021;8:615978. 10.3389/fmed.2021.615978 PMC8079659 33937277

[8] Younossi ZM, Loomba R, Anstee QM, Rinella ME, Bugianesi E, Marchesini G, et al. Diagnostic modalities for nonalcoholic fatty liver disease, nonalcoholic steatohepatitis, and associated fibrosis. Hepatology. 2018;68(1):349–60. 29222917 10.1002/hep.29721 PMC6511364

[9] Wu L, Shen Y, Li F. Non-invasive diagnosis of liver fibrosis: A review of current imaging modalities. Gastroenterología Y Hepatología (English Edition). 2020;43(4):211–21. 10.1016/j.gastrohep.2019.11.009 32089376

[10] Zhang C-Y, Liu S, Yang M. Treatment of liver fibrosis: Past, current, and future. World J Hepatol. 2023;15(6):755. 37397931 10.4254/wjh.v15.i6.755 PMC10308286

[11] Younossi ZM, Loomba R, Rinella ME, Bugianesi E, Marchesini G, Neuschwander-Tetri BA, et al. Current and future therapeutic regimens for nonalcoholic fatty liver disease and nonalcoholic steatohepatitis. Hepatology. 2018;68(1):361–71. 29222911 10.1002/hep.29724 PMC6508084

[12] Allinovi M, De Chiara L, Angelotti ML, Becherucci F, Romagnani P. Anti-fibrotic treatments: a review of clinical evidence. Matrix Biol. 2018;68:333–54. 29654884 10.1016/j.matbio.2018.02.017

[13] Fang L, Murphy AJ, Dart AM. A clinical perspective of anti-fibrotic therapies for cardiovascular disease. Front Pharmacol. 2017;8:262882. 10.3389/fphar.2017.00186 PMC5382201 28428753

[14] Yoon YJ, Friedman SL, Lee YA, editors. Antifibrotic therapies: where are we now? Seminars in liver disease. Thieme Medical; 2016. 10.1055/s-0036-1571295 26870935

[15] Rahmani AH, Alsahli MA, Aly SM, Khan MA, Aldebasi YH. Role of Curcumin in disease prevention and treatment. Adv Biomedical Res. 2018;7(1):38. 10.4103/abr.abr_147_16 PMC5852989 29629341

[16] Chen Y, Lu Y, Lee RJ, Xiang G. Nano encapsulated curcumin: and its potential for biomedical applications. Int J Nanomed. 2020:3099–120. 10.2147/IJN.S210320 PMC7200256 32431504

[17] Wu W, Shen J, Banerjee P, Zhou S. Water-dispersible multifunctional hybrid nanogels for combined Curcumin and photothermal therapy. Biomaterials. 2011;32(2):598–609. 20933280 10.1016/j.biomaterials.2010.08.112

[18] Yallapu MM, Jaggi M, Chauhan SC. Curcumin nanoformulations: a future nanomedicine for cancer. Drug Discovery Today. 2012;17(1–2):71–80. 21959306 10.1016/j.drudis.2011.09.009 PMC3259195

[19] Wong KE, Ngai SC, Chan KG, Lee LH, Goh BH, Chuah LH. Curcumin nanoformulations for colorectal cancer: a review. Front Pharmacol. 2019;10:152. 10.3389/fphar.2019.00152. 30890933 10.3389/fphar.2019.00152 PMC6412150

[20] Peng S, Li Z, Zou L, Liu W, Liu C, McClements DJ. Improving Curcumin solubility and bioavailability by encapsulation in saponin-coated Curcumin nanoparticles prepared using a simple pH-driven loading method. Food Funct. 2018;9(3):1829–39. 10.1039/c7fo01814b. 29517797 10.1039/c7fo01814b

[21] Sivani BM, Azzeh M, Patnaik R, Pantea Stoian A, Rizzo M, Banerjee Y. Reconnoitering the therapeutic role of Curcumin in disease prevention and treatment: lessons learnt and future directions. Metabolites. 2022;12(7):639. 35888763 10.3390/metabo12070639 PMC9320502

[22] Laurindo LF, de Carvalho GM, de Oliveira Zanuso B, Figueira ME, Direito R, de Alvares Goulart R, et al. Curcumin-based nanomedicines in the treatment of inflammatory and Immunomodulated diseases: an evidence-based comprehensive review. Pharmaceutics. 2023;15(1):229. 36678859 10.3390/pharmaceutics15010229 PMC9861982

[23] Gorabi AM, Hajighasemi S, Kiaie N, Rosano GM, Sathyapalan T, Al-Rasadi K, et al. Anti-fibrotic effects of Curcumin and some of its analogues in the heart. Heart Fail Rev. 2020;25:731–43. 31512150 10.1007/s10741-019-09854-6

[24] Chan AW, Tetzlaff JM, Gøtzsche PC, Altman DG, Mann H, Berlin JA, et al. SPIRIT 2013 explanation and elaboration: guidance for protocols of clinical trials. BMJ (Clinical Res ed). 2013;346:e7586. 10.1136/bmj.e7586. 10.1136/bmj.e7586 PMC3541470 23303884

[25] Barr RG, Wilson SR, Rubens D, Garcia-Tsao G, Ferraioli G. Update to the society of radiologists in ultrasound liver elastography consensus statement. Radiology. 2020;296(2):263–74. 10.1148/radiol.2020192437. 32515681 10.1148/radiol.2020192437

[26] Chung M, Baird GL, Weiss KE, Beland MD. 2D shear wave elastography: measurement acquisition and reliability criteria in noninvasive assessment of liver fibrosis. Abdom Radiol (New York). 2019;44(10):3285–94. 10.1007/s00261-019-02183-0. 10.1007/s00261-019-02183-0 31435762

[27] Matsukuma K, Yeh MM. Practical Guide, Challenges, and pitfalls in liver fibrosis staging. Surg Pathol Clin. 2023;16(3):457–72. 10.1016/j.path.2023.04.002. 37536882 10.1016/j.path.2023.04.002

[28] Ebrahimi-Mousavi S, Alavian SM, Sohrabpour AA, Dashti F, Djafarian K, Esmaillzadeh A. The effect of daily consumption of probiotic yogurt on liver enzymes, steatosis and fibrosis in patients with nonalcoholic fatty liver disease (NAFLD): study protocol for a randomized clinical trial. BMC Gastroenterol. 2022;22(1):102. 10.1186/s12876-022-02176-2. 35255811 10.1186/s12876-022-02176-2 PMC8899796

[29] Craig CL, Marshall AL, Sjöström M, Bauman AE, Booth ML, Ainsworth BE, et al. International physical activity questionnaire: 12-country reliability and validity. Med Sci Sports Exerc. 2003;35(8):1381–95. 10.1249/01.Mss.0000078924.61453.Fb. 12900694 10.1249/01.MSS.0000078924.61453.FB

[30] Eslamparast T, Poustchi H, Zamani F, Sharafkhah M, Malekzadeh R, Hekmatdoost A. Synbiotic supplementation in nonalcoholic fatty liver disease: a randomized, double-blind, placebo-controlled pilot study. Am J Clin Nutr. 2014;99(3):535–42. 10.3945/ajcn.113.068890. 24401715 10.3945/ajcn.113.068890

[31] Roehlen N, Crouchet E, Baumert TF. Liver fibrosis: mechanistic concepts and therapeutic perspectives. Cells. 2020;9(4). 10.3390/cells9040875 10.3390/cells9040875 PMC7226751 32260126

[32] Gupta SC, Patchva S, Aggarwal BB. Therapeutic roles of curcumin: lessons learned from clinical trials. AAPS J. 2013;15:195–218. 23143785 10.1208/s12248-012-9432-8 PMC3535097

[33] Panahi Y, Rahimnia AR, Sharafi M, Alishiri G, Saburi A, Sahebkar A. Curcuminoid treatment for knee osteoarthritis: A randomized double-blind placebo‐controlled trial. Phytother Res. 2014;28(11):1625–31. 24853120 10.1002/ptr.5174

[34] Naksuriya O, Okonogi S, Schiffelers RM, Hennink WE. Curcumin nanoformulations: a review of pharmaceutical properties and preclinical studies and clinical data related to cancer treatment. Biomaterials. 2014;35(10):3365–83. 24439402 10.1016/j.biomaterials.2013.12.090

