# Supplementary material for: Efficacy of the nano curcumin supplementation on fibrosis, steatosis, inflammatory and metabolic status of liver fibrosis patients with non-alcoholic fatty liver origin: a study protocol for a double-blind randomized controlled trial
Source: BMC Nutr. 2026 Feb 2;12:45. doi: 10.1186/s40795-026-01252-0 (PMC12955327; doi:10.1186/s40795-026-01252-0)
Supplement: Supplementary file 1 — Supplementary Material 1. [file 40795_2026_1252_MOESM1_ESM.docx]

**«هوالشافی»**

**رضايت نامه شرکت در طرح** بررسی تاثیر مکمل یاری نانوکورکومین بر روی بیماران مبتلا به فیبروز کبدی با منشاء کبد چرب غیر الکلی می باشد.

**آقاي/ خانم محترم**

بدين وسيله از شما جهت شركت در پژوهش فوق‌الذکر دعوت به عمل مي‌آيد. اطلاعات مربوط به این پژوهش در این برگه خدمتتان ارائه شده است و شما برای شرکت یا عدم شرکت در این پژوهش آزاد هستید.

شما مجبور به تصميم گيري فوري نيستيد و برای تصميم گيري در اين باره مي‌توانيد سوالات خود را از تيم پژوهشي بپرسيد و با هر فردي که مایل باشید مشورت نماييد. قبل از امضاي اين رضايت نامه مطمئن شويد كه متوجه تمامي اطلاعات اين فرم شده‌ايد و به تمام سوالات شما پاسخ داده شده است.

**مجري پژوهش**

1. من مي‌دانم كه اهداف اين پژوهش عبارتند از: تعیین تاثیر مکمل یاری نانوکورکومین بر روی بیماران مبتلا به فیبروز کبدی با منشاء کبد چرب غیر الکلی می باشد.
2. من مي­دانم که شرکت من در اين پژوهش کاملاً داوطلبانه است و مجبور به شرکت در اين پژوهش نيستم.

به من اطمينان داده شد که اگر حاضر به شركت در اين پژوهش نباشم، از مراقبت‌هاي معمول تشخيصي و درماني محروم نخواهم شد و رابطه درماني من با مركز درماني و پزشك معالجم دچار اشكال نمي‌شود.

1. من مي‌دانم كه حتي پس از موافقت با شركت در پژوهش مي‌توانم هر وقت كه بخواهم، پس از اطلاع به مجري، از پژوهش خارج شوم و خروج من از پژوهش باعث محرومیت از دریافت خدمات درمانی معمول برای من نخواهد شد.
2. نحوه‌ي همکاري اينجانب در اين پژوهش به اين‌صورت است:

هر شرکت کننده در این مطالعه ابتدا از لحاظ شرایط ورود به مطالعه سنجیده می شود و پرسشنامه اطلاعات دموگرافیک، پرسشنامه یادداشت خوراک و پرسشنامه فعالیت فیزیکی از آنها تکمیل می گردد. در صورت داشتن شرایط ورود به مطالعه، 50 بیمار مبتلا به فیبروز کبدی با منشاء کبد چرب غیر الکلی وارد مطالعه می شوند، این افراد در دو گروه دريافت کننده کپسول نانوکورکومین( گروه مداخله ) و دارونما (گروه شاهد) قرار مي گيرند. هر شرکت کننده بسته به گروهی که در آن قرار دارد، تعداد دو عدد کپسول( حاوی40 میلی گرم نانوکورکومین یا دارونما) دریافت خواهد کرد. از افراد شرکت کننده در هر گروه 14 سی سی نمونه ناشتای خون (بعد از 12-10 ساعت ناشتایی) جهت بررسی فاکتور های خونی در ابتدا و انتهای مطالعه گرفته می شود. همکاری در این طرح حدود 4 ماه طول می کشد. در کل هر شرکت کننده در این مطالعه 7 بار مراجعه خواهد کرد، و آنهم برای خونگیری، گرفتن فیبر و اسکن، بادی آنالیز و گرفتن کپسول ها می باشد. در ابتدا و انتهاي مطالعه وزن ، قد ، دور کمر، سنجش ترکیب بدنی و نمونه هاي خوني براي همه شرکت کنندگان اندازه گيري مي شود.

1. منافع احتمالي شرکت اينجانب در اين مطالعه به اين شرح است:

در پژوهش فوق درمان مورد نظر براي بيماران احتمالا شامل کاهش عوارض ناشی از بيماری در گروه دریافت کننده نانوکورکومین می باشد. با شرکت در این طرح اگر مشکل دیگری در فرد شرکت کننده تشخیص داده شود حتماً به متخصصین امر برای انجام مشاوره معرفی خواهد شد.

هزینه های این طرح من الجمله انجام آزمایشات خون و فیبر و اسکن کبدی و بررسی سنجش ترکیب بدنی یا بادی آنالیز به صورت رایگان می باشد و در طول مدت حضور در طرح، بیماران از درمان های استاندارد خود محروم نخواهند ماند.

1. آسيب‌ها و عوارض احتمالي شرکت در اين مطالعه به اين شرح است: این طرح خطر احتمالی برای شرکت کنندگان ندارد، با این وجود در صورت بروز هر گونه صدمات احتمالي، محققين پژوهش غرامت لازم را پرداخت مي نمايند و تمام هزينه هايي که ممکن است شما در حين اجراي مطالعه ملزم به پرداخت آن شويد، توسط پژوهشگران بازپرداخت مي شود.
2. در صورت عدم تمایل به شرکت در مطالعه روش معمول درمانی برای من ارائه خواهد شد که منافع و عوارض آن به این شرح است: اگر مشکوک به بیماری خاصی باشم به من پیشنهاد می شود به متخصص مربوطه مراجعه کنم
3. من مي­دانم كه دست اندر كاران اين پژوهش، كليه اطلاعات مربوط به من را نزد خود به صورت محرمانه نگه‌ داشته و فقط اجازه دارند فقط نتايج كلي و گروهي اين پژوهش را بدون ذکر نام و مشخصات اينجانب منتشر كنند.
4. می­دانم که كميته اخلاق در پژوهش با هدف نظارت بر رعایت حقوق اينجانب مي‌تواند به اطلاعات من دسترسي داشته باشد.
5. من مي‌دانم كه هيچ‌ يک از هزينه‌ هاي انجام مداخلات پژوهشي به شرح ذيل بر عهده من نخواهد بود.
6. خانم حدیث گرامی جهت پاسخگويي به اينجانب معرفي شد و به من گفته شد تا هر وقت مشكلي يا سوالي در رابطه با شركت در پژوهش مذكور پيش آمد با ايشان در ميان بگذارم و راهنمايي بخواهم.

آدرس و شماره تلفن ثابت و همراه ايشان به شرح به من ارائه شد:

- **آدرس:**
- **تلفن ثابت: ...........................................................................**
- **تلفن همراه:09036745467**

1. من مي‌دانم كه اگر در حين و بعد از انجام پژوهش هر مشكلي اعم از جسمي و روحي به علت شرکت در اين پژوهش براي من پيش آمد درمان عوارض، و هزينه‌هاي آن و غرامت مربوطه بر عهده مجري خواهد بود.
2. من مي­دانم اگر اشکال يا اعتراضي نسبت به دست اندركاران يا روند پژوهش دارم مي­توانم با كميته اخلاق در پژوهش دانشگاه علوم پزشكي یزد به آدرس: یزد- میدان شهید دکتر باهنر- سازمان مرکزی دانشگاه علوم پزشکی و خدمات بهداشتی درمانی شهید صدوقی یزد، تماس گرفته و مشکل خود را به صورت شفاهي يا كتبي مطرح نمايم.
3. اين فرم اطلاعات و رضايت آگاهانه در دو نسخه تنظيم شده و پس از امضا يک نسخه در اختيار من و نسخه ديگر در اختيار مجري قرار خواهد گرفت.

اينجانب موارد فوق ‌الذکر را خواندم و فهميدم و بر اساس آن رضايت آگاهانه خود را براي شركت در اين پژوهش اعلام مي‌کنم.

امضاي شركت كننده

اينجانب ……………… خود را ملزم به اجراي تعهدات مربوط به مجري در مفاد فوق دانسته و متعهد مي‌گردم در تأمين حقوق و ايمني شركت كننده در اين پژوهش تلاش نمايم.

مهر و امضاي مجري پژوهش
